# Role of microglia in blood pressure and respiratory responses to acute hypoxic exposure in rats

**DOI:** 10.1186/s12576-022-00848-y

**Published:** 2022-10-13

**Authors:** Masashi Yoshizawa, Isato Fukushi, Kotaro Takeda, Yosuke Kono, Yohei Hasebe, Keiichi Koizumi, Keiko Ikeda, Mieczyslaw Pokorski, Takako Toda, Yasumasa Okada

**Affiliations:** 1 Department of Pediatrics, Faculty of Medicine, University of Yamanashi, Yamanashi, Japan; 2 Clinical Research Center, Murayama Medical Center, Tokyo, Japan; 3 Faculty of Health Sciences, Aomori University of Health and Welfare, Aomori, Japan; 4 Faculty of Rehabilitation, School of Health Sciences, Fujita Health University, Toyoake, Japan; 5 Department of Pediatrics, Fujiyoshida Municipal Hospital, Yamanashi, Japan; 6 Institute of Innovative Research, Homeostatic Mechanism Research Unit, Tokyo Institute of Technology, Yokohama, Japan; 7 Institute of Health Sciences, Opole University, Opole, Poland

**Keywords:** Acute hypoxia, Blood pressure telemetry, Cardiorespiratory regulation, Hypoxic ventilatory response, Microglia

## Abstract

Microglia modulate cardiorespiratory activities during chronic hypoxia. It has not been clarified whether microglia are involved in the cardiorespiratory responses to acute hypoxia. Here we investigated this issue by comparing cardiorespiratory responses to two levels of acute hypoxia (13% O_2_ for 4 min and 7% O_2_ for 5 min) in conscious unrestrained rats before and after systemic injection of minocycline (MINO), an inhibitor of microglia activation. MINO increased blood pressure but not lung ventilation in the control normoxic condition. Acute hypoxia stimulated cardiorespiratory responses in MINO-untreated rats. MINO failed to significantly affect the magnitude of hypoxia-induced blood pressure elevation. In contrast, MINO tended to suppress the ventilatory responses to hypoxia. We conclude that microglia differentially affect cardiorespiratory regulation depending on the level of blood oxygenation. Microglia suppressively contribute to blood pressure regulation in normoxia but help maintain ventilatory augmentation in hypoxia, which underscores the dichotomy of central regulatory pathways for both systems.

## Background

Acute hypoxia is sensed mainly by peripheral chemoreceptors, elevates arterial blood pressure, and increases ventilation [1–4]. Previous studies focused mainly on neurons, regarding the cellular mechanisms of central hypoxic cardiorespiratory regulation [5–8]. In recent years, the involvement of glial cells, particularly astrocytes, in the hypoxic regulation of cardiorespiratory function has attracted attention [9–19]. Microglia, another type of glial cells, are known to evoke pro/anti-inflammatory responses in the central nervous system and contribute to synaptic plasticity [20–22]. For example, microglia play an important role in learning and memory [23] and various pathological conditions, such as allodynia, i.e., the sustenance of nociceptive disorders [24]. Regarding the role of microglia in hypoxic responses of the cardiorespiratory system, both in vivo and in vitro studies reported their augmenting or suppressing action [20, 25–34]. In those studies, hypoxia was usually loaded to animals over a relatively long time of a few hours to several days in sustained or chronic intermittent hypoxia routines. Both chronic and acute intermittent hypoxia also modulate sympathetic and respiratory neuronal network interactions [35–43]. However, the role of microglia in cardiorespiratory responses to hypoxic exposure of minutes’ duration, which sufficiently induces cardiorespiratory excitation, has not been fully elucidated. Based on this background, we aimed to investigate whether microglia would be involved in the modulation of cardiorespiratory responses to acute hypoxia by simultaneously measuring blood pressure and ventilation in conscious rats.

## Methods

### Animals

We used Wistar rats for this study (*n* = 6, aged 23–26 weeks). Only male rats were used to avoid possible sex differences in microglial gene expression within the cardiorespiratory network [44] and the influence of the menstrual cycle. Experiments were performed with the approval of the Ethics Committee for Animal Experiments of the Murayama Medical Center in Tokyo (approval numbers: 12–2) and complied with the Guidelines for Care and Use of Laboratory Animals released by the National Research Council of the National Academies (8th edition, revised 2011) and with the Guiding Principles for Care and Use of Animals of the Physiological Society of Japan. The animals were purchased from Sankyo Labo Service (Tokyo, Japan), individually housed in plastic cages under a constant room temperature (23 ~ 24 °C), 50–60% relative humidity, and a 12-h light–dark cycle with access to standard commercial chow and water ad libitum. All efforts were made to minimize animal suffering and to reduce the number of animals used.

### Preparation for blood pressure measurement

To measure arterial blood pressure, we used a telemetry system. Rats were anesthetized with isoflurane inhalation followed by injection of pentobarbital sodium (50 mg/kg, i.p.). The abdominal cavity was opened by a midline incision to expose the aorta. A telemeter transmitter (TRM 54P, Kaha Sciences, Auckland, New Zealand) was placed in the cavity, and the transducer cable (length 9 cm, outer diameter 500 μm) with a pressure sensor at its tip (tip’s outer diameter 660 μm) was inserted into the aorta in the caudo-rostral direction from the level 2–3 mm rostral to the common iliac arteries. The transducer cable was fixed to the aortic surface with tissue adhesive (Vetbond, 3 M, Saint Paul, MN). An antibiotic (cefazolin, 50 mg/kg) dissolved in saline was applied before closing the abdomen. The surgery was conducted aseptically. The rats were allowed to recover from surgery for at least 1 week.

### Recording of blood pressure and heart rate

Unrestrained rats were placed in a recording chamber of the whole-body plethysmograph (see details below). Blood pressure signals were continuously transmitted to a receiving and processing unit (TR181/TR190, Kaha Sciences) positioned beneath the recording chamber of the plethysmograph. Raw blood pressure signals were digitized at a 2 kHz sampling rate with an A/D converter (PL 3504 Power Lab 2/26, AD Instruments, Colorado Springs, CO) and stored in a PC with LabChart7 software (AD Instruments) for offline analysis. The mean arterial pressure (MAP; mmHg) was computed from the blood pressure waveforms and the pulse rate was counted using LabChart7. The pulse rate precisely coincided with the heart rate (HR; beats/min) obtained in the electrocardiographic recording in preliminary experiments. Thus, HR was used hereafter in this work.

### Recording of ventilation

Ventilatory variables were measured by whole-body plethysmography. A recording chamber (volume 3.77 L) was placed inside a transparent acrylic box (size 30 × 30 × 30 cm). The chamber temperature was maintained constant at 25 °C throughout the experiment. The air in the recording chamber was continuously suctioned with a constant flow generator that supplied fresh air into the chamber. To calculate the respiratory flow, the pressure difference between the recording and reference chambers was measured with a differential pressure transducer (TPF100, EMMS), connected to an amplifier (AIU060, Information & Display Systems, Bordon, UK), and was bandpass filtered at 0.1–20 Hz. The signal was integrated to obtain tidal volume (${\dot {\text V}}$T; mL/kg body weight) for each respiratory cycle, which was then averaged. Respiratory rate (RR; breaths/min) was counted. Minute ventilation (${\dot {\text V}}$E; mL/kg/min) was calculated as ${\dot {\text V}}$T × RR. The oxygen concentration was monitored with an oxygen analyzer incorporating a polarographic sensor (Respina IH 26, San-ei, Tokyo, Japan) and was adjusted to the desired level by controlling the flows of nitrogen and air blown into the acrylic box. Respiratory flow and oxygen concentration signals were simultaneously digitized at a 400 Hz sampling rate with an A/D converter (Power Lab 4/26, AD Instruments) and stored in a PC with LabChart7 software (AD Instrument).

Blood pressure and ventilatory signals were measured by two researchers using two independent recording systems. The two recordings were conducted simultaneously.

### Inhibition of microglial activity

Blood pressure and ventilatory measurements were conducted before and after administration of minocycline (MINO), a selective suppressor of stimulus-induced microglia activation [45–47]. The interval between the measurements before and after MINO administration was at least 1 week. MINO suppressed microglial proliferation and activation by inhibiting p38 MAPK, existing in the active form only in microglia [29, 46], inflammatory cytokine secretion, toll-like receptor 2 (TLR2) expression [48], and nuclear translocation of NF-κB [49]. It easily crosses the blood–brain barrier due to its high lipid solubility. In the present study, MINO (Fuji Pharma, Tokyo Japan) was diluted in physiological saline and neutralized with sodium hydroxide solution to adjust pH to 7.4 (final concentration 11.8 mg/mL) and was administered in a dose of 25 mg/kg/day, i.p. for 3 consecutive days before the experiment, with the last dose at least 3 h before measurements [34, 47].

### Loading of hypoxia

For acclimatization, each rat was placed in the recording chamber for 60 min before the onset of measurements. Then, after taking the baseline blood pressure and ventilation recordings in room air for 2 min, the oxygen concentration in the chamber was rapidly lowered to 13% (mild hypoxia) by blowing nitrogen into the acrylic box under monitoring the intra-chamber oxygen concentration. After 4 min, the chamber gas was returned to room air, and recording continued for at least a 12-min post-hypoxia recovery phase, further referred to as “recovery.” Variables were continuously recorded through the pre-hypoxic baseline, hypoxic, and post-hypoxic recovery phases. After we confirmed that animals fully recovered from the hypoxic stress and the variables stabilized after mild hypoxia, severe hypoxia (7% O_2_) was induced for 5 min and the measurements were repeated similarly. To evaluate the magnitudes of hypoxia-induced changes in MAP, HR, and VE, hypoxia-baseline and recovery-baseline differences (ΔMAP, ΔHR, and ΔVE) were calculated.

### Statistical analysis

Data were presented as means ± SD. A two-way analysis of variance (ANOVA) was conducted to examine the effects of hypoxia on MAP, ΔMAP, HR, ΔHR, RR, ${\dot {\text V}}$T, ${\dot {\text V}}$E, and Δ${\dot {\text V}}$E﻿ with a two- ‘drug’ condition (control-MINO) and a six-oxygen phase or four-oxygen phase for ΔMAP, ΔHR, and Δ${\dot {\text V}}$E﻿ as within-subject factors. The oxygen phases included mean values just before hypoxia (0–2-min baseline), the end of hypoxia (5–7 min in mild and 6–8 min in severe hypoxia), and recovery (16–18 min in both mild and severe hypoxia). The reason we adopted the mean values instead of peak values was that these physiological signals fluctuate and spontaneously vary to some extent and thus values at single time points do not adequately represent changes. The Greenhouse–Geisser adjustment was used to correct for violations of sphericity whenever necessary. We applied the Bonferroni correction for multiple comparisons in post-hoc tests. A *p* < 0.05 defined a statistically significant difference. The analysis was performed using SPSS 27.0 (IBM, Armonk, NY).

## Results

### Circulatory responses to hypoxia and MINO treatment

#### Mean arterial blood pressure (MAP)

**Fig. 1:**
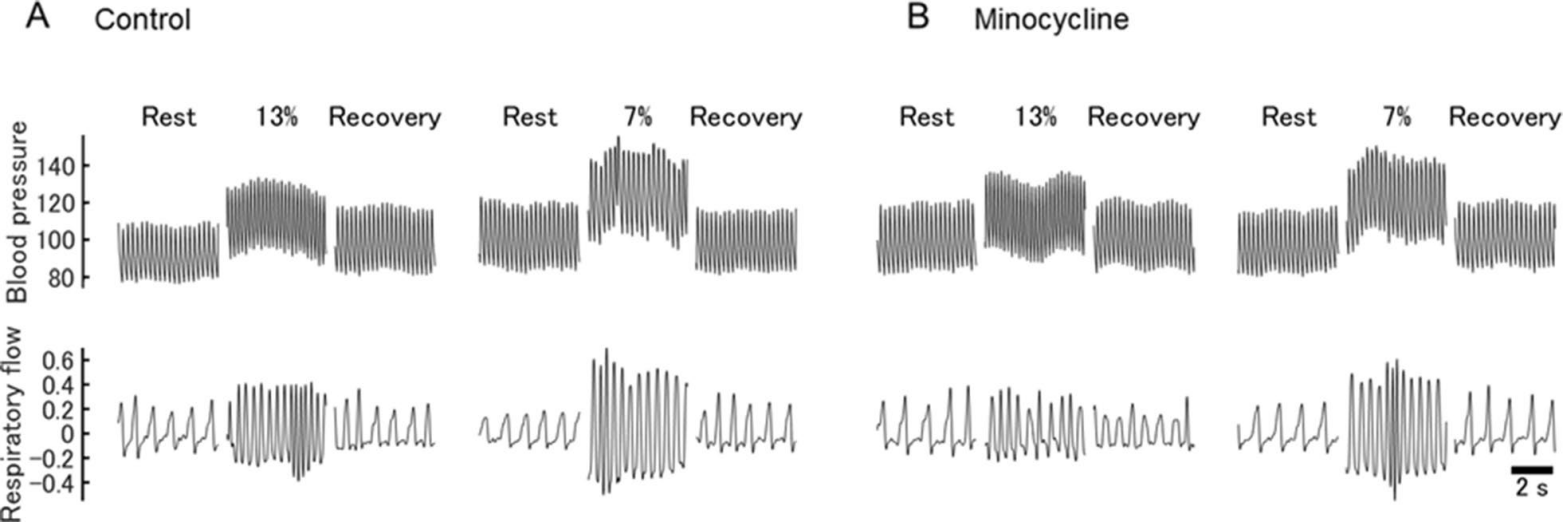
Effects of minocycline (MINO) on blood pressure and ventilatory responses to mild (13% O_2_) and severe (7% O_2_) hypoxia. Representative traces of blood pressure and respiratory flow signals (inspiration upward). **A** Control condition (MINO-untreated). **B** MINO-treated condition

**Fig. 2:**
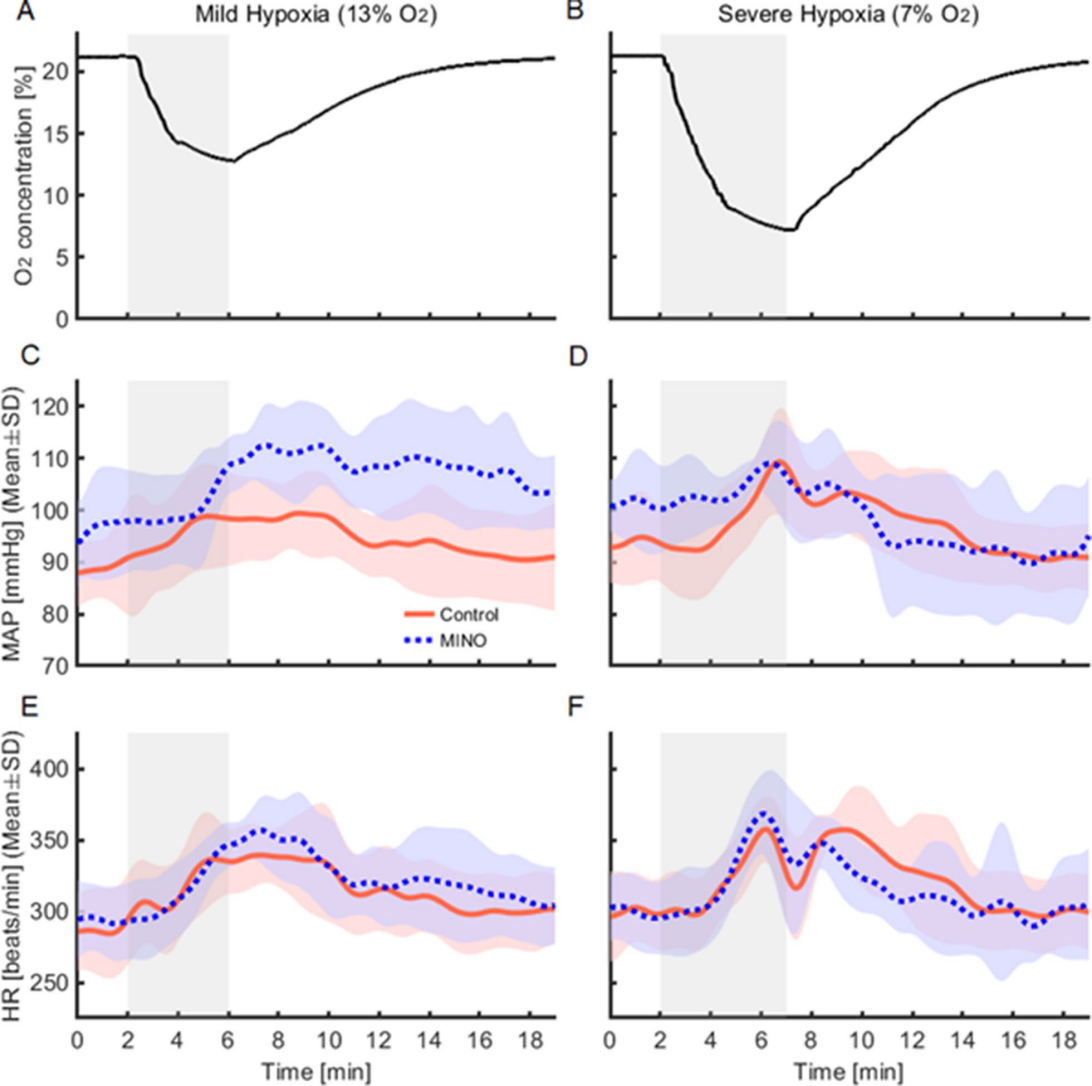
Effects of minocycline (MINO) on mean arterial pressure (MAP) and heart rate (HR) responses to mild (13% O_2_) and severe (7% O_2_) hypoxia (shaded areas). **A**, **B** Representative timelines of the intrachamber oxygen concentrations during mild and severe hypoxia (shaded periods). **C,** **D** Time courses of MAP in the control (MINO-untreated) and MINO-treated rats during mild and severe hypoxia. **E,** **F** Time courses of HR in the control (MINO-untreated) and MINO-treated rats during mild and severe hypoxia

**Fig. 3:**
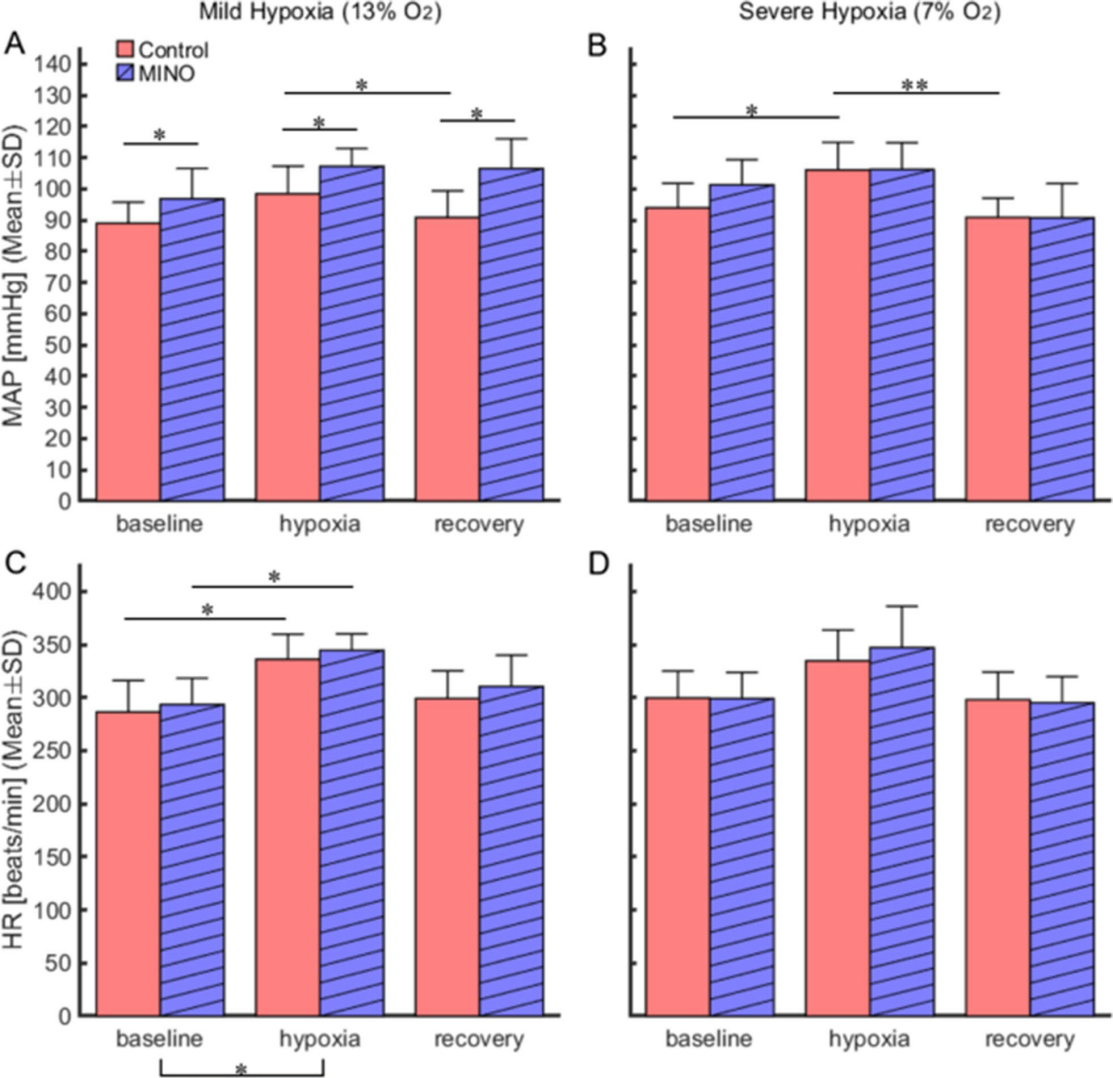
Effects of minocycline (MINO) on mean arterial pressure (MAP) and heart rate (HR) responses to mild (13% O_2_) and severe (7% O_2_) hypoxia. Mean values were compared among the baseline (0–2 min before hypoxia loading), end-hypoxia (5–7 min in mild hypoxia and 6–8 min in severe hypoxia), and recovery (16–18 min) phases. **A,** **B** Comparison of MAP among three oxygen phases during mild and severe hypoxia. MAP values in the mild hypoxia condition were higher in the MINO-treated condition compared to the control (MINO-untreated) in all oxygen phases. **C,** **D** Comparison of HR values among three oxygen phases during mild and severe hypoxia. * *p* < 0.05, ** *p* < 0.01

At baseline normoxia, MAP was higher in MINO-treated than MINO-untreated rats (96.8 ± 9.7 vs 89.0 ± 6.8 mmHg,* p* < 0.05) in the mild hypoxia protocol. A similar tendency was in severe hypoxia, although the difference was insignificant (Figs. 1, 2, 3). In MINO-untreated rats, both mild and severe hypoxia increased MAP. In mild hypoxia, the increase was higher than that in recovery (*p* < 0.05), and in severe hypoxia it was higher than those at baseline and recovery (*p* < 0.05 and *p* < 0.01, respectively). In MINO-treated rats, MAP increased at both hypoxic levels significantly more over the increase in the untreated ones (*p* < 0.05). There was a significant interaction between MINO conditions and oxygen levels (*F*(5, 25) = 2.86, *p* < 0.05) (Figs. 2, 3).

**Fig. 4:**
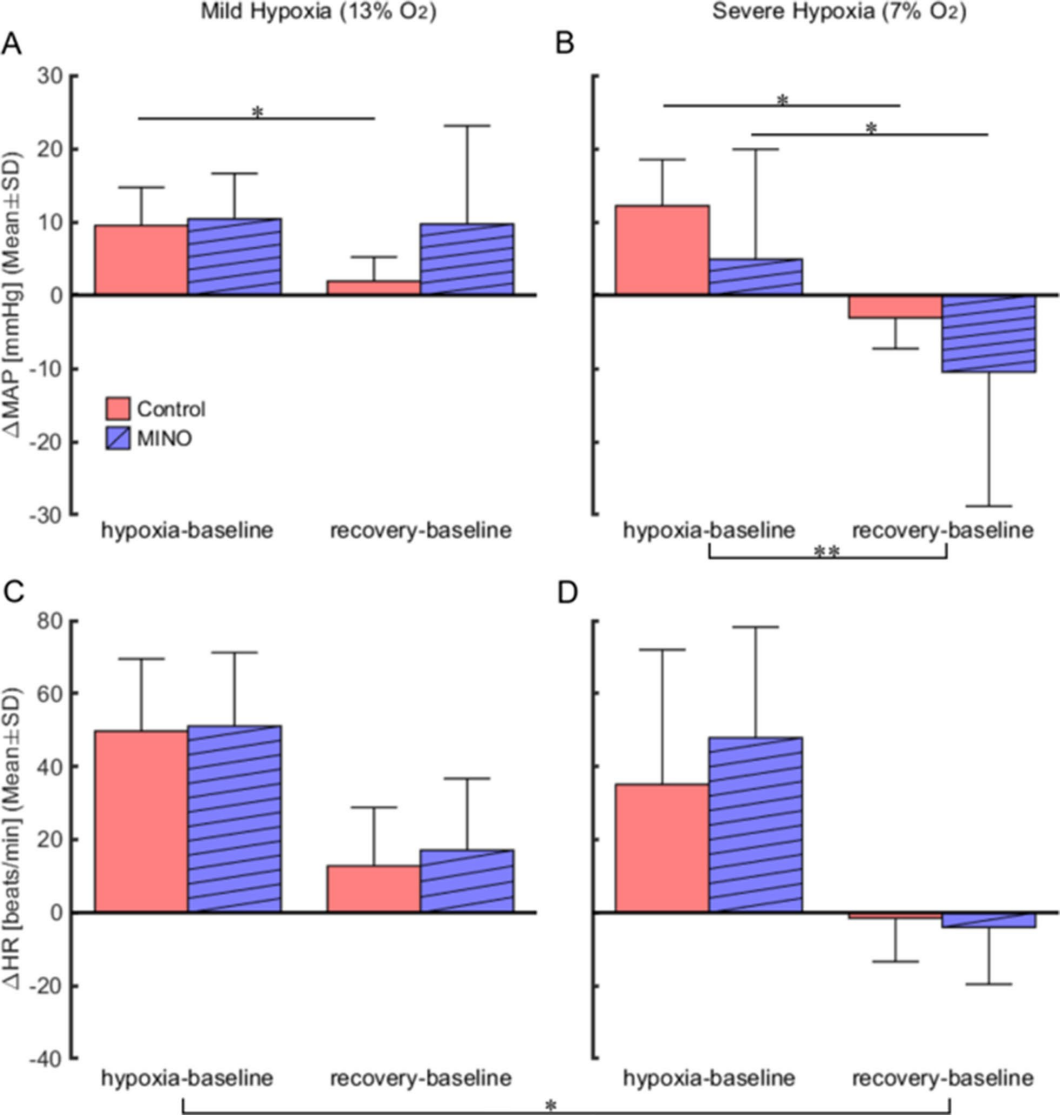
Effects of minocycline (MINO) on mean blood pressure (MAP) and heart rate (HR) responses to mild (13% O_2_) and severe (7% O_2_) hypoxia. To evaluate response magnitudes, ΔMAP and ΔHR were calculated for the hypoxia-baseline and recovery-baseline differences. ΔMAP and ΔHR were compared between the control (MINO-untreated) and MINO-treated conditions. There was the oxygen main effect on blood pressure responses in severe hypoxia, and ΔMAP increased during hypoxia and decreased during recovery. The post-hoc test revealed that there was not a significant difference between any pairs. * *p* < 0.05, ** *p* < 0.01

A comparative evaluation of differences in MAP increases showed that ΔMAP was higher in severe hypoxia than in recovery (a significant main effect of oxygen levels (*F*(3,15) = 6.63, *p* < 0.01). However, the main effect between before and after MINO (*F*(1,5) = 0.54, *p* = 0.495) and the interaction between MINO-treatment and oxygen levels (*F*(3,15) = 1.34, *ε*_GG_ = 0.37, *p* = *0*.302) were insignificant (Fig. 4).

#### Heart rate (HR)

At baseline normoxia, HR did not differ between MINO-treated and MINO-untreated rats (Figs. 1, 2). HR increased in mild hypoxia compared to the baseline level; *p* < 0.05 (calculations included both MINO-untreated and treated conditions). The main effect of oxygen levels was significant (*F*(5,25) = 12.30, *ε*_GG_ = 0.39, *p* < 0.01). However, neither the interaction between MINO-treatment and oxygen levels (*F*(5,25) = 1.281, *p* = *0*.303) nor the main effect between before and after MINO was significant (*F*(1,5) = 0.80, *p* = 0.413) (Figs. 2, 3).

For ΔHR, there was a significant main effect of oxygen levels (*F*(3,15) = 9.83, *p* < 0.010), but it was detected only in mild hypoxia and recovery from severe hypoxia; *p* < 0.01 (calculations included both MINO-untreated and treated conditions). Neither the interaction between MINO-treatment and oxygen levels (*F*(3,15) = 0.56, *p* = *0*.562) nor the main effect between before and after MINO were significant (*F* (1,5) = 1.17, *p* = 0.329) (Fig. 4).

### Ventilatory responses to hypoxia and MINO treatment

**Fig. 5:**
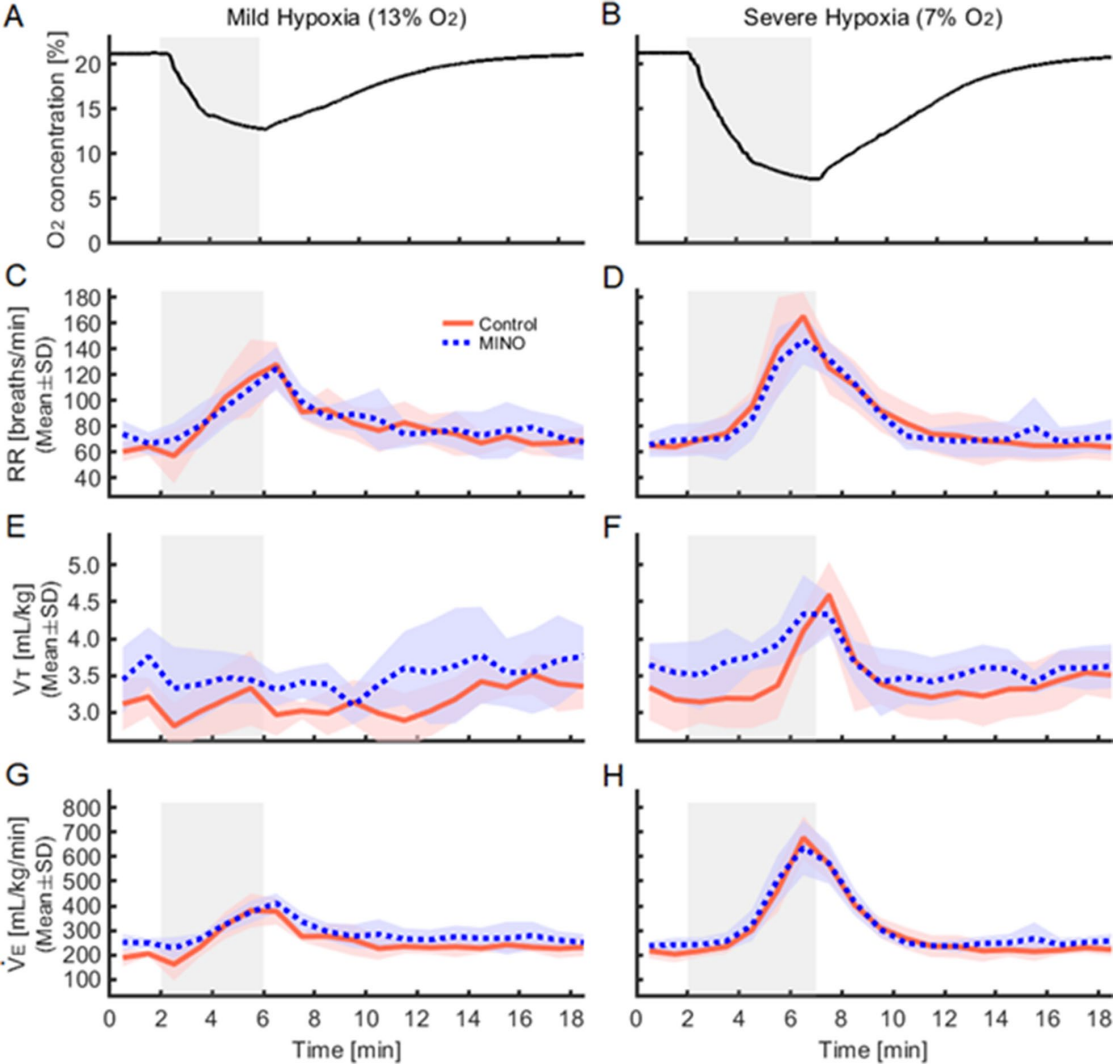
Effects of minocycline (MINO) on ventilatory responses to mild (13% O_2_) and severe (7% O_2_) hypoxia. **A,** **B** Representative timelines of the intrachamber oxygen concentrations during mild and severe hypoxia (shaded periods). **C,** **D** Time courses of respiratory rate (RR) in the control (MINO-untreated) and MINO-treated rats during mild and severe hypoxia. **E,** **F** Time courses of tidal volume (${\dot {\text V}}$T) in the control (MINO-untreated) and MINO-treated conditions during mild and severe hypoxia. **G,** **H** Time courses of minute ventilation (${\dot {\text V}}$E) in the control (MINO-untreated) and MINO-treated conditions during mild and severe hypoxia loading

**Fig. 6:**
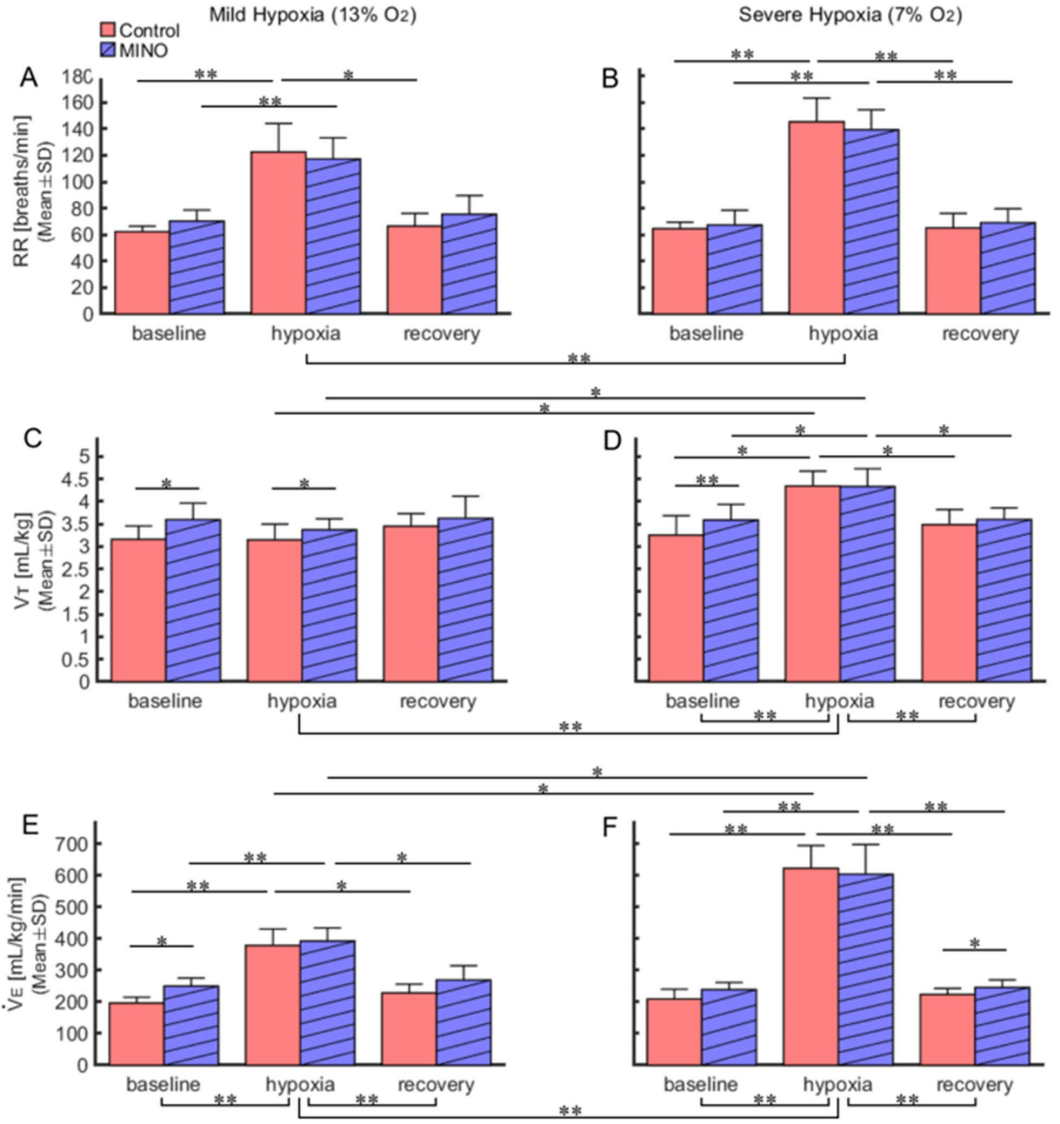
Effects of minocycline (MINO) on ventilatory responses to mild (13% O_2_) and severe (7% O_2_) hypoxia. Mean values were compared among the baseline (0–2 min before hypoxia), end-hypoxia (5–7 min in mild hypoxia and 6–8 min in severe hypoxia), and recovery (16–18 min) phases. **A,** **B** Comparison of respiratory rate (RR) among three oxygen phases during mild and severe hypoxia. RR was higher during hypoxia than in other phases (for details, see Results). **C,** **D** Comparison of tidal volume (${\dot {\text V}}$T) among three oxygen phases during mild and severe hypoxia. **E,** **F** Comparison of minute ventilation (${\dot {\text V}}$E﻿) among three oxygen phases during mild and severe hypoxia. In both mild and severe hypoxia, ${\dot {\text V}}$E was larger during hypoxia than during baseline and recovery (*p* < 0.01). In severe hypoxia, ${\dot {\text V}}$E﻿ was larger than that in mild hypoxia (*p* < 0.01). * *p* < 0.05, ** *p* < 0.01

Ventilatory variables responded sluggishly to MINO injection at baseline normoxia. They tended, on average, to increase but changes failed to reach significance. Hypoxia, expectedly, caused hyperventilation that was mostly driven by RR increase; the effect was distinctly potentiated at the stronger stimulus level (Figs. 5, 6).

#### Respiratory rate (RR)

RR consistently increased in hypoxia both before and after MINO treatment. In mild hypoxia in MINO-untreated rats, RR was higher than those at baseline and recovery (*p* < 0.01 and *p* < 0.05, respectively). The same was true for severe hypoxia (*p* < 0.01). In mild hypoxia in MINO-treated rats, RR was higher than that at baseline (*p* < 0.01), and in severe hypoxia, it was higher than those at baseline and recovery (*p* < 0.01). RR increased significantly more in severe than mild hypoxia (*p* < 0.01). There was a significant interaction between the two MINO conditions and oxygen levels (*F*(5, 25) = 2.62, *p* < 0.05). However, RR failed to differ significantly before and after MINO conditions at either level of oxygen (Figs. 1, 5, 6).

#### Tidal volume (${\dot {\text V}}$T)

${\dot {\text V}}$T﻿ was higher in severe hypoxia than those at baseline and recovery, and its increase was the highest in severe hypoxia; *p* < 0.01 (calculations included both MINO-untreated and treated conditions) (Figs. 1, 5, 6). There was no significant interaction between the MINO conditions and oxygen levels (*F*(5,25) = 1.52, *p* = 0.219). However, there was a significant main effect between the two MINO conditions (*F*(1,5) = 7.38, *p* < 0.05), meaning that ${\dot {\text V}}$T was, as overall, higher in MINO-treated than MINO-untreated conditions. In addition, there was a significant main effect of oxygen levels (*F*(5,25) = 9.83, *ε*_GG_ = 0.43, *p* < 0.001).

#### Minute ventilation (${\dot {\text V}}$E﻿)

${\dot {\text V}}$E was higher in both mild and severe hypoxia when compared with the baseline and recovery levels and it was the highest in severe hypoxia; *p* < 0.01 (calculations included both MINO-untreated and treated conditions). There was no significant interaction between the MINO conditions and oxygen levels (*F* (5,25) = 2.10, *p* = 0.099). Neither was there a significant main effect between the two MINO conditions (*F* (1,5) = 2.07, *p* = 0.210). However, there was a significant main effect of oxygen levels (*F*(5,25) = 125.67, *ε*_GG_ = 0.26, *p* < 0.001) (Figs. 1, 5, 6).

**Fig. 7:**
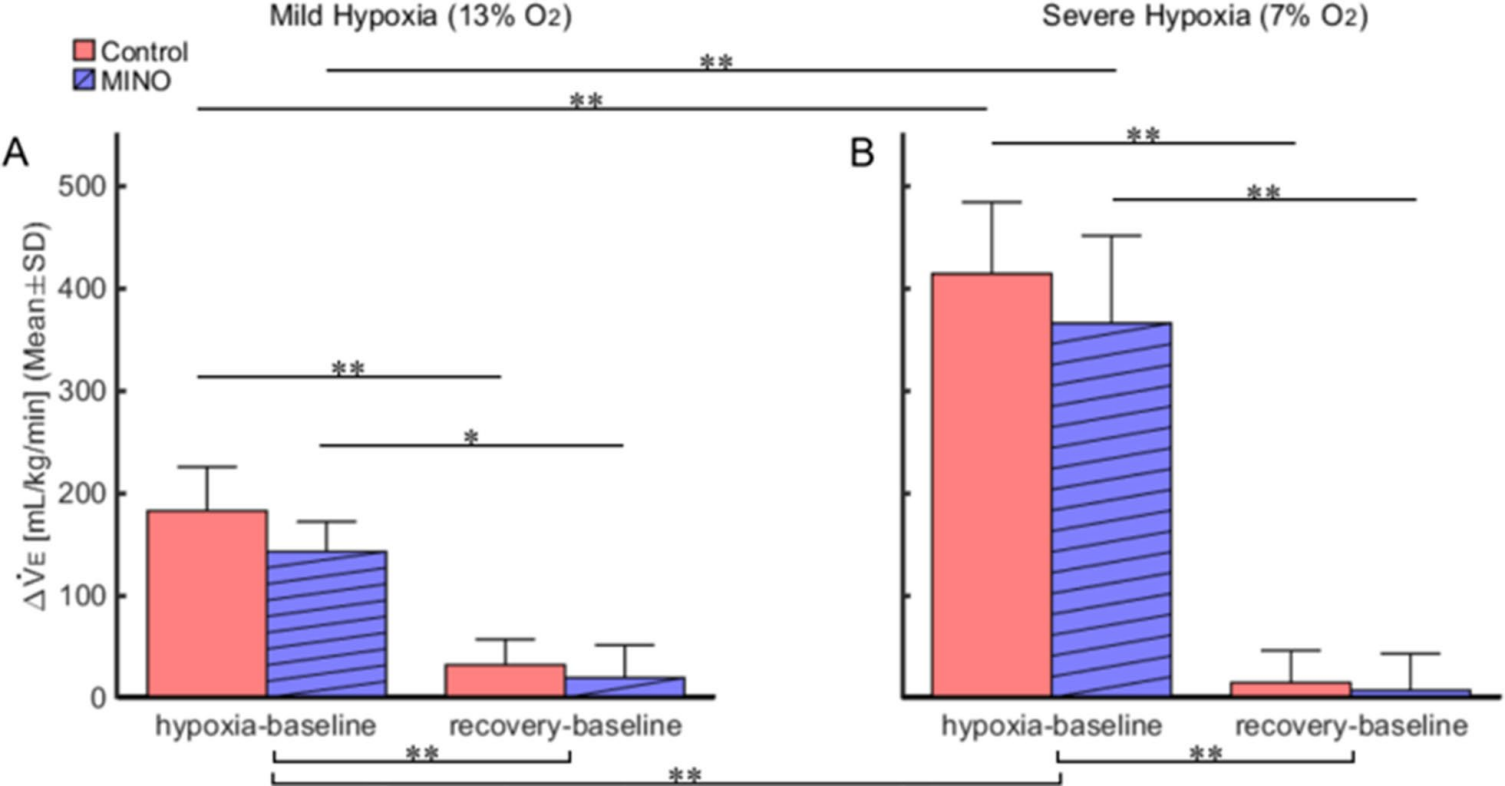
Effects of minocycline (MINO) on ventilatory responses to mild (13% O_2_) (**A**) and severe (7% O_2_) (**B**) hypoxia. To evaluate response magnitudes of minute ventilation, Δ${\dot {\text V}}$e was calculated for the hypoxia-baseline and recovery-baseline differences. Δ${\dot {\text V}}$e was significantly larger in hypoxia than in recovery (** *p* < 0.01). There was a significant main effect between the two MINO conditions (*F*(1,5) = 16.78, *p* < 0.01) meaning that Δ${\dot {\text V}}$e was, as overall, smaller in the MINO treated than untreated condition across all oxygen phases. The hypoxia-baseline Δ${\dot {\text V}}$e﻿ in the control MINO-untreated condition tended to be higher than that in the MINO-treated one in both mild and severe hypoxia, but the differences were statistically insignificant. * *p* < 0.05, ** *p* < 0.01

As for Δ${\dot {\text V}}$E, there was no significant interaction between the MINO conditions and oxygen levels (*F*(3,15) = 0.76, *p* = *0*.532). There was a significant main effect between the two MINO conditions (*F*(1,5) = 16.78, *p* < 0.01) meaning that Δ${\dot {\text V}}$E was, as overall, smaller in the MINO treated than untreated condition across all oxygen phases. The hypoxia-baseline Δ${\dot {\text V}}$E in the control MINO-untreated condition tended to be higher than that in the MINO-treated one in both mild and severe hypoxia, but the differences were statistically insignificant. There also was a significant main effect of oxygen phases (*F*(3,15) = 104.21, *p* < 0.001) (Fig. 7).

## Discussion

In the present study, using a pharmacologic inhibitor of microglia activation, minocycline (MINO), we investigated the involvement of microglia in blood pressure and ventilatory responses to acute exposures to mild and severe hypoxia in unrestrained conscious rats. Although hypoxia was loaded only for several minutes, it was sufficient to induce augmentation of the cardiorespiratory function. In the control MINO-untreated condition, hypoxia elevated MAP and increased HR, as previously reported [50], although the responses did not follow the stimulus severity-dependency. In MINO-treated condition, MAP increased more than in MINO-untreated one across all oxygen phases in mild hypoxia. However, MINO did not affect the magnitude of hypoxia-induced blood pressure elevation (ΔMAP) or HR increase (ΔHR).

In contrast to cardiovascular responses, hypoxia increased ventilation in a stimulus-dependent manner in both MINO-untreated and treated conditions. MINO tended to increase ${\dot {\text V}}$T across all oxygen phases in both mild and severe hypoxia, although the treatment per se did not affect RR or ${\dot {\text V}}$E﻿. However, as assessed by Δ${\dot {\text V}}$E﻿, MINO treatment tended to attenuate hypoxia-induced ventilatory augmentation.

These results suggest that microglia contributed to the blood pressure homeostasis by mitigating its elevation in the normoxic condition but their role in the acute hypoxia-induced blood pressure elevation could not be convincingly shown. On the other side, the microglia’s contribution to ventilation in the normoxic condition was insignificant, but it appeared to play an active role in the augmentation of ventilatory responses to acute hypoxia. These findings indicate that the suppressive/augmenting action of microglia is differential concerning blood pressure and ventilatory regulations. It suppresses blood pressure in the normal state but augments ventilation in the hypoxic state.

Microglia are resident immune cells in the brain. They are activated by various stress stimulations and change their shape from ‘ramified’ to ‘reactive’ with retracted branching processes. Regarding the possible morphologic changes of microglial cells, a much longer time than minutes’ exposure applied in the present study, approaching at least 1 h, is required to notice shorter and fewer cellular outgrowths in the cardiorespiratory brain region in rats [31]. However, recent studies show that microglial functional changes do not necessarily require morphologic counterparts and may rapidly arise upon various types of stimulation. In mouse and rat spinal cord slices, perfusion with a lipopolysaccharide (LPS)-mimetic agent causes nearly an instant activation of toll-like receptor 4 (TLR4) in 1 min, increasing intracellular Ca^2+^ in microglia with a peak delay of 3.8 min [51]. Likewise, in mouse hippocampal slices, activation of microglia by LPS increases the frequency of spontaneous excitatory postsynaptic currents in neurons within minutes [52]. In focal injuries of the mouse cortex, surrounding microglia respond in the first minute of post-ablation with tips of cellular processes being enlarged in the vicinity of injury [53]. Furthermore, microglia respond with large, generalized Ca^2+^ transients damaging individual cortical neurons with a latency of only 0.4–4.0 s, which is much more rapid than ever considered [54]. Even in the resting brain with a ‘ramified’ phenotype, microglia are highly and constantly dynamic with motile processes and protrusions [55–57]. Thus, it could be expected that acute hypoxia lasting for several minutes might cause functional activation of microglia, affecting the cardiorespiratory function.

Several studies have reported the association between hypoxic cardiorespiratory responses and microglia. Microglia are involved in the autonomic modulation by regulating inflammatory cytokines in the paraventricular nucleus of the hypothalamus and rostral ventrolateral medulla during acute hypoxic stimulation [21, 58]. In steady-state hypoxia, suppression of microglia activation inhibits the expression of inflammatory markers in the autonomic brainstem nuclei during 3-h hypoxic stimulation and attenuates hypoxic ventilatory augmentation during 10-min hypoxic stimulation in rats [32]. It has also been shown that hypoxia-induced blood pressure elevation is suppressed by MINO treatment in rats during 24-h sustained hypoxia [34].

In the present study, inhibition of microglia activation elevated blood pressure but did not affect ventilation in the pre-hypoxic baseline phase, which suggests that microglia play a role in the regulation of blood pressure by mitigating its elevation but not ventilation in the normoxia. That is in line with the reports showing that microglia have an anti-inflammatory and neuron-suppressing M2 phenotype at rest [22, 59]. On the other hand, inhibition of microglia suppressed the hypoxia-induced ventilatory increase, indicating that microglia play a role in the augmentation of ventilation in acute hypoxia. These findings may be explained by hypoxia's rapid activation of microglia in the respiratory center and the phenotype change from the anti-inflammatory neuron-suppressing M2 to the pro-inflammatory neuron-stimulating M1 [22, 59]. The major mechanism of microglia activation by hypoxia consists of increased expression of potassium channels, e.g., Kiv1.1 and Kiv1.2, which facilitate the secretion of inflammatory cytokines from microglia [56, 60]. In addition, hypoxia can activate microglia by upregulation of TLR4 at the mRNA and protein levels [61] and purinergic receptor expression [62].

The putative role of microglia in augmenting hypoxic ventilation is akin to the presumptive role of astrocytes in hypoxia sensing by the brain tissue [9–19]. It has been reported that activated microglia release ATP that stimulates astrocytic purinergic receptors and increases the excitatory postsynaptic current frequency in neurons through a metabotropic glutamate receptor mechanism [52]. The action of microglia in augmenting ventilatory responses to acute hypoxia demonstrated in the present study may be attributed to the positive interaction of microglia and astrocytes upstream to neurons [28, 52].

Concerning neural plasticity in the cardiorespiratory control, we have reported that astrocytes mediate post-hypoxic persistent respiratory augmentation (PHRA), i.e., sustained potentiation of breathing for a time after the cessation of acute hypoxic exposure. The breathing potentiation was suppressed by inhibition of astrocytic activation in mice [12]. Contrary to that report, post-hypoxic persistent cardiorespiratory augmentation was not observed in the present study. This discrepancy may be attributed to the species difference. Alternatively, longer hypoxic exposure might be required to induce post-hypoxic PHRA and blood pressure elevation.

There have been several reports concerning the role of microglia for cardiorespiratory neural plasticity. Microglia activated by LPS attenuate phrenic long-term facilitation following acute intermittent hypoxia in rats [25–27]. Stokes et al. [31] reported that hypoxia activates both microglia and astrocytes. The activated glial cells mediate ventilatory acclimatization to hypoxia as chronic hypoxia augmented responsiveness to acute hypoxia persists upon return to normoxia; the phenomenon is suppressed by blockade of microglial activation [31]. Other studies also show a role of microglia for intermittent hypoxia, resembling sleep-disordered breathing, or steady-state hypoxia. In intermittent hypoxia, underlain by reshaped carotid body chemosensory reflexes, microglia participate in enhanced sympathetic activation persisting after breathing normalization [3, 63, 64]. It has been reported that cytokines released by microglia in intermittent hypoxia cause a long-term potentiation of cardiopulmonary function, contributing to neuroplasticity and respiratory adaptation to hypoxia [20]. The role of microglia for respiratory plasticity appears complex and may be either facilitating or inhibiting depending on experimental conditions [33]. Microglia also appear to shape synaptic plasticity in pathophysiological conditions [21, 22, 24], and may be involved with cardiorespiratory disorders in humans, such as hypertension, sleep apnea, sudden infant death syndromes, and others. Further studies are needed using alternative study designs, e.g., genetically modified animals, to explore the role of microglia in the cardiorespiratory function [65].

## Conclusions

In synopsis, the study showed that microglia contributed to cardiovascular but not ventilatory control by counteracting the arterial blood pressure augmentation in the resting normoxic condition. On the other hand, microglia appeared to contribute to respiratory but not cardiovascular control by augmenting ventilatory responses to acute hypoxia. Taken together, the present and other reports suggest the modulatory role of microglia be mediated by an interaction between microglia and astrocytes upstream to neurons. Differences in shaping the cardiovascular and ventilatory regulation by these two non-neuronal brain cells require further exploration using alternative study designs.

## Data Availability

The data sets used in the present study are available from the corresponding author upon reasonable request.

## Abbreviations

ANOVA: Analysis of variance
HR: Heart rate
LPS: Lipopolysaccharide
MAP: Mean arterial pressure
NF-κB: Nuclear factor-kappa B
p38 MAPK: P38 mitogen-activated protein kinase
MINO: Minocycline
PHRA: Post-hypoxic persistent respiratory augmentation
RR: Respiratory rate
TLR2: Toll-like receptor 2
TLR4: Toll-like receptor 4
$${\dot {\text V}} $$e: Minute ventilation
$${\dot {\text V}} $$t: Tidal volume
